# Gut microbiota and the prevalence and incidence of renal stones

**DOI:** 10.1038/s41598-022-07796-y

**Published:** 2022-03-08

**Authors:** Han-Na Kim, Jae Heon Kim, Yoosoo Chang, Dongmin Yang, Kwan Joong Joo, Young-Sam Cho, Heung Jae Park, Hyung-Lae Kim, Seungho Ryu

**Affiliations:** 1 Medical Research Institute, Kangbuk Samsung Hospital, Sungkyunkwan University School of Medicine, Seoul, Republic of Korea; 2 Department of Clinical Research Design and Evaluation, SAIHST, Sungkyunkwan University, Seoul, Republic of Korea; 3 Department of Urology, Soonchunhyang University Seoul Hospital, Soonchunhyang University Medical College, Seoul, Republic of Korea; 4 Center for Cohort Studies, Total Healthcare Center, Kangbuk Samsung Hospital, Sungkyunkwan University School of Medicine, Seoul, Republic of Korea; 5 Department of Occupational and Environmental Medicine, Kangbuk Samsung Hospital, Sungkyunkwan University School of Medicine, Samsung Main Building B2, 250, Taepyung-ro 2ga, Jung-gu, Seoul, 04514 Republic of Korea; 6 Department of Urology, Kangbuk Samsung Hospital, Sungkyunkwan University School of Medicine, Seoul, Republic of Korea; 7 Department of Biochemistry, College of Medicine, Ewha Womans University, Seoul, Republic of Korea

**Keywords:** Renal calculi, Microbiology

## Abstract

The role of the gut microbiome in the development of renal stone diseases has not been well characterized*.* This study focused on the taxonomic and functional profiles of gut microbiomes according to the prevalence and incidence of nephrolithiasis. Stool samples from 915 Korean adults were collected at baseline. Participants were followed for a median of 4.0 years. We evaluated the biodiversity of the gut microbiota and taxonomic profiles associated with nephrolithiasis status, using 16S rRNA gene sequencing. Nephrolithiasis status was categorized into three groups: control (no-stone at both baseline and follow-up visits), incidental nephrolithiasis, and prevalent nephrolithiasis. Compared to the control and incidental nephrolithiasis, the prevalent nephrolithiasis showed a reduced evenness in alpha diversity. Nephrolithiasis was associated with a reduced abundance of some key taxa involved in short-chain fatty acid production. Moreover, the abundance of *Bifidobacterium,* which possess oxalate-degrading ability, was higher in the control. Conversely, there was no significant difference in the bacterial composition between the incidental and prevalent nephrolithiasis. In our study with repeated nephrolithiasis measurements, prevalent renal stones were associated with an altered gut microbiota composition compared to the control. Besides the known oxalate degradation pathway, other functional pathways inferred in this study require further investigation.

## Introduction

Nephrolithiasis, also known as kidney stone disease, is a common disease worldwide, with a prevalence of 5–10%. With a continuous increase in its prevalence and incidence, nephrolithiasis poses a considerable global health and economic burden^1–3^. Supersaturation of urine by constituents such as calcium, oxalate, or uric acid is the most widely accepted mechanism of nephrolithiasis^4,5^. However, its pathogenesis and risk factors are not yet fully understood.

Recently, contribution of the gut bacteria to the pathophysiological gastrointestinal-renal axis has received increasing attention. Gastrointestinal microbiota has been suggested to play a role in oxalate metabolism, one of major proportion of nephrolithiasis, possibly by affecting the intestinal absorption of oxalate and renal stone formation^6–8^. However, research on this topic has mainly focused on *Oxalobacter formigenes* that utilizes oxalate as a principal energy substrate^9^. Its existence in feces has been correlated with a diminished renal excretion of oxalate and a decreased risk of stone recurrence^7^. Multiple previous studies have failed to identify a significant role of *O. formigenes* in nephrolithiasis, because isolation of *O. formigenes* from feces is not feasible^9^. However, recent analytical methods, such as 16S ribosomal RNA gene sequencing and shotgun metagenomics, have facilitated delineation of the gastrointestinal microbiome. At present, only five human studies, which have shown inconsistent findings, have evaluated the association between nephrolithiasis and the gastrointestinal microbiome^10–14^. All five of these studies included patients with symptomatic renal stone disease and were limited by small sample sizes and cross-sectional associations with single-time measurements.

The present study aimed to examine the association between the prevalence and incidence of renal stones and the gut microbiota in a relatively large-scale study of 915 participants.

## Methods

### Study participantss

This prospective cohort study enrolled participants who underwent comprehensive annual or biennial examinations at the Kangbuk Samsung Hospital Healthcare Screening Center in South Korea^15^ between June 2014 and September 2014. A total of 1463 Korean adults aged 23 to 78 years who agreed to participate in this study and provided stool samples at Kangbuk Samsung Hospital Healthcare Screening Center^16^ were included. Fecal samples were obtained only at baseline. Abdominal ultrasonography is a routine part of a health checkup program.

**Figure 1:**
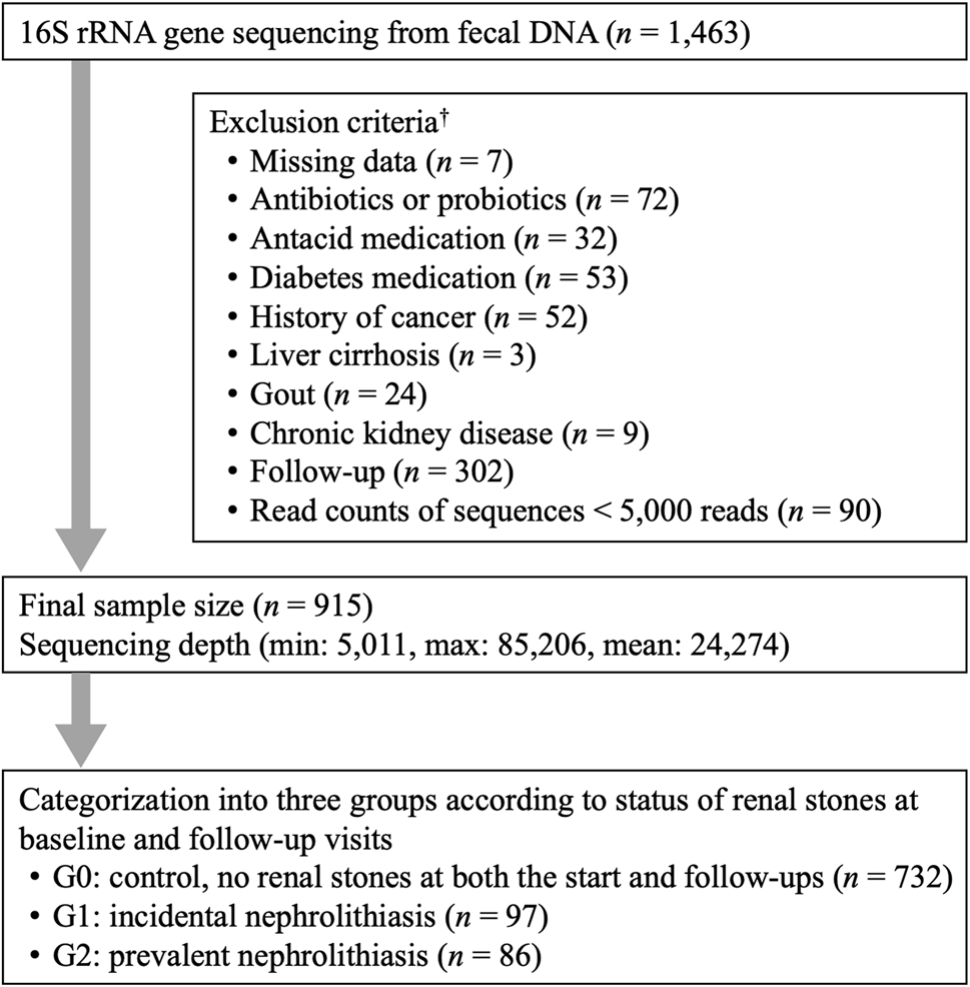
Inclusion and exclusion criteria for study participants. ^†^Some individuals met several exclusion criteria.

Participants who met any of the exclusion criteria were not included in the analysis (Fig. 1). We excluded 548 participants based on the following criteria: missing data (*n* = 7); use of antibiotics within 6 weeks prior to enrollment or probiotics within 4 weeks prior to enrollment (*n* = 72); history of cancer (*n* = 52); current use of antacid medication (*n* = 32); use of diabetes medication (*n* = 53); history of liver cirrhosis or identification of liver cirrhosis on an ultrasonography (*n* = 3); gout (n = 24); chronic kidney disease (*n* = 9); and samples with less than 5000 sequences (see below for further details) (*n* = 90). As the changes in the renal stone status over time were the focus of this study, participants with no follow-up visits by December 31, 2017, were also excluded (*n* = 302). None of the patients had a history of stomach, colon, kidney, or bladder surgery. Some individuals met more than one of the exclusion criteria. In the final analysis, a total of 915 participants were included. The characteristics of the study participants and the selection process are provided in detail in the Supplementary Methods. Dietary consumption was assessed using a 103-item self-administered food frequency questionnaire (FFQ) developed for use in Korea^17^. This validated FFQ was designed to measure usual food consumption during the previous year. Data were collected during the health checkup.

The present prospective cohort study was conducted according to a protocol approved by the Institutional Review Board of Kangbuk Samsung Hospital (KBSMB 2013-01-245-12 and 2020-09-003). Written informed consent was obtained from all participants included in this study. Demographic, laboratory, and other clinical data, along with stool samples, were collected according to the ethical guidelines compliant with the Declaration of Helsinki.

### Renal stone and group definition

Abdominal ultrasonography was performed during the first and follow-up visits by experienced radiologists who were blinded to the study aim using a Logic Q700 MR with a 3.5 MHz transducer (Logic Q700 MR; GE, Milwaukee, WI, USA; LOGIQ E9, GE, Madison, WI, USA). All participants underwent abdominal ultrasonography in the supine or right-lateral position for both kidneys. Nephrolithiasis was diagnosed when hyperechoic structures causing acoustic shadowing were observed in the collecting system on ultrasonography^18^. Based on the renal stone status at the initial and subsequent visits, patients were categorized into the following groups: G0, no renal stones (control), those without renal stones at the initial and subsequent visits; G1, incidental renal stones, those without renal stones initially but with renal stones at the follow-up visits; and G2, prevalent renal stones, those with renal stones at the beginning of the experiment. The median follow-up period was 4.0 years (interquartile range, 2.0–5.0 years, maximum 5.5 years); each participant was followed from baseline until the development of renal stones or the last follow-up examination, whichever came first.

### Sample collection, DNA extraction, and 16S rRNA gene sequencing

Fresh fecal samples were collected from each volunteer, in a sterile container, immediately frozen at –20 °C, and stored at –70 °C within 24 h until further manipulation. Total DNA was extracted from the stool samples within 1 month of storage using the MOBio PowerSoil DNA Isolation Kit (MO BIO Laboratories, Carlsbad, CA, USA) according to the manufacturer’s instructions.

To amplify and sequence the V3–V4 hypervariable region of the 16S rRNA gene, specific fusion primers were used (Illumina, San Diego, CA, USA). Libraries were pooled for sequencing, using the full complement of Nextera XT indices, and sequenced on the Illumina MiSeq platform (Illumina, San Diego, CA, USA) according to the manufacturer’s instructions^19^.

### Microbial profiling and statistical analysis

Demultiplexed sequences were processed using DADA2, which is a plugin of the QIIME2 package (version 2017.11, https://qiime2.org )^20^ and the low-quality regions of the sequences and chimeras were removed. After denoising with DADA2, amplicon sequence variants (ASVs) were generated and regarded as 100% operational taxonomic units. Then, we constructed a feature table containing the counts of each unique sequence in each sample. We filtered out features present in only one sample, based on the suspicion that they did not represent actual biological diversity, and were attributed to polymerase chain reaction or sequencing errors.

The biodiversity of the samples (⍺-diversity) was calculated using the number of ASVs observed in each sample, the Shannon index, which accounts for both evenness and richness, Faith’s phylogenetic diversity^21^, and Pielou’s evenness. As some of the variables were not normally distributed, the Kruskal–Wallis test was used to compare the differences among groups. The dissimilarity between samples (β-diversity) was calculated using the UniFrac distance^22^ to estimate dissimilarities among group members by incorporating the phylogenetic distances between ASVs. Unweighted and weighted UniFrac distances were calculated to determine the presence or absence and abundance of ASVs, respectively. Non-phylogenetic β-diversity indices, such as the Bray–Curtis dissimilarity index^23^ were also used for the abundance data. We used pairwise permutational multivariate analysis of variance with 999 random permutations to test the significance of the differences among groups. The microbial community composition was depicted using a principal coordinate analysis plot. All tests were corrected for multiple tests. Basic statistical analyses were performed using R Studio (version 1.3.1073; R Foundation for Statistical Computing, Vienna, Austria), and microbial diversity plots were plotted using the “ggplot2” R package (version 3.3.2).

Taxonomy was assigned to ASVs using a pre-trained naïve Bayes classifier and the q2-feature-classifier plugin against the Silva database (version r138.1) of the 16S rRNA sequence database in the QIIME2 package (version 2020.8, https://qiime2.org). To investigate any significant differences in the relative taxa abundances from the phylum to genus levels among groups, we used two statistical tools from R (version 4.0.2): analysis of composition of microbiomes (ANCOM, v2.1)^24^ and generalized linear models implemented in multivariate association with linear models (MaAsLin2). After adjusting for age, sex and BMI, we compared the abundance of taxa among G0, G1, and G2 in a pairwise manner. ANCOM compares the relative abundance of taxa among groups by the log-ratio of the abundance of each taxon to that of the remaining taxa, one at a time. The final significance was expressed in the empirical distribution of *W* at each taxonomic level. We used the taxa-wise false discovery rate (FDR) option with the significance level set to FDR < 0.05 to generate *W* statistics and a threshold of 0.6 for declaring a significant association. For the MaAsLin2 models, G0 was set as the reference group and compared with G1 and G2. The coefficient values and *p*-values that passed the significance threshold (Benjamini–Hochberg FDR *p*-value < 0.05) are represented in the MaAsLin2 analyses. Linear discriminant analysis effect size (LEfSe) was used to detect potential nephrolithiasis-specific bacterial markers^25^. LEfSE utilizes a two-step statistical analysis including the nonparametric Kruskal–Wallis sum-rank test to identify bacterial taxa, which differ significantly in abundance between groups. This is followed by linear discriminant analysis to estimate the effect size of each differentially abundant feature. Only taxa with a linear discriminant analysis score (log_10_) greater than three (*p* < 0.05) were considered significantly enriched.

### Prediction of metagenome functions

For functional inferences of the microbial community, we conducted a Phylogenetic Investigation of Communities by Reconstruction of Unobserved States (PICRUSt2) (v2.2.0-b)^26^ analysis of the ASVs according to the instructions published at https://github.com/picrust/picrust2/wiki. PICRUSt2 predictions were based on the gene family enzyme classification (EC) numbers (as of January 21, 2016). We generated a metabolic pathway database (MetaCyc) using pathway abundance predictions from the EC-based gene family predictions^27^. We also inferred Kyoto Encyclopedia of Genes and Genomes (KEGG) pathways levels by using the last publicly available mapping of KEGG orthologs to pathways (which is from around 2011)^28,29^. The predicted functional pathways were compared among the groups using statistical analysis of taxonomic and functional profiles (STAMP) version 2.1.3^30^. Statistical differences in the pathways were tested using Welch’s t-test with a Benjamini–Hochberg FDR correction (*q* < 0.05) to adjust for multiple tests.

## Results

### Participants demographics

Baseline characteristics of the enrolled participants according to renal stone status are shown in Table 1. Of the 915 participants (mean age: 40.21 years, men 64.70%), 732 were in no renal stone (G0) group, 97 in incidental renal stone (G1) group, and 86 in the prevalent renal stone (G2) group. The G2 group was the oldest on an average and had the highest prevalence of hypertension among the three groups. There were no significant differences in morbidities, including diabetes, obesity, metabolic syndrome, and nutritional intake (Supplementary Table S1) among the three groups.

**Table 1:** Demographic and clinical characteristics of the study participants according to renal stone status.

Characteristics	Overall	Stone category			*P*
		G0 (No-stone)	G1 (Incidental stones)	G2 (Prevalent stones)	
Number (male %)^§^	915 (64.7%)	732 (64.34%)	97 (67.01%)	86 (65.12%)	0.872
Age at baseline (year)^†^	40.21 (± 7.89)	39.77 (± 7.79)	40.89 (± 6.87)	43.19 (± 9.16)	< 0.001
BMI (kg/m^2^)^†^	23.57 (± 3.08)	23.56 (± 3.15)	23.69 (± 3.14)	23.50 (± 2.44)	0.907
Waist circumference (cm)^†^	82.24 (± 8.80)	82.17 (± 9.02)	82.42 (± 8.52)	82.58 (± 7.08)	0.900
Systolic BP (mmHg)^†^	109.41 (± 13.45)	109.26 (± 13.53)	109.68 (± 14.41)	110.38 (± 11.63)	0.747
Diastolic BP (mmHg)^†^	70.97 (± 10.16)	70.76 (± 10.14)	71.92 (± 11.35)	71.70 (± 8.89)	0.453
Glucose (mg/dL)^†^	94.17 (± 11.94)	93.91 (± 10.84)	94.23 (± 16.38)	96.33 (± 14.67)	0.208
Total cholesterol (mg/dL)^†^	198.11 (± 32.97)	197.95 (± 32.96)	196.23 (± 32.55)	201.56 (± 33.74)	0.529
LDL-C (mg/dL)^†^	120.35 (± 29.71)	120.21 (± 29.58)	119.16 (± 31.43)	122.85 (± 29.03)	0.681
HDL-C (mg/dL)^†^	56.92 (± 14.10)	56.78 (± 14.05)	56.73 (± 14.77)	58.35 (± 13.84)	0.614
Triglycerides (mg/dL)^‡^	100.00 (± 72.00)	102.50 (± 73.00)	95.00 (± 61.00)	96.50 (± 64.00)	0.912
hsCRP (mg/dL)^†^	0.91 (± 1.89)	0.92 (± 1.79)	0.82 (± 1.55)	1.01 (± 2.90)	0.807
Current smoker (N, %)^§^	157 (17.16%)	115 (15.71%)	24 (24.74%)	18 (20.93%)	0.024
Alcohol intake (> 20 mg; N, %)^§^	210 (22.95%)	174 (22.75%)	22 (22.68%)	22 (25.58%)	0.801
Hypertension (N, %)^§^	107 (11.69%)	77 (10.52%)	12 (12.37%)	18 (20.93%)	0.017
Diabetes (N, %)^§^	22 (2.64%)	16 (2.19%)	2 (2.06%)	4 (4.65%)	0.359
Obesity (N, %)^§^	242 (26.45%)	199 (27.19%)	23 (23.71%)	20 (23.26%)	0.598
Metabolic syndrome (N, %)^§^	115 (12.57%)	92 (12.57%)	12 (12.37%)	11 (12.79%)	0.996

BMI, body mass index; BP, blood pressure; LDL-C, low-density lipoprotein cholesterol; HDL-C, high-density lipoprotein cholesterol; and hsCRP, high-sensitivity C-reactive protein.

Participants were compared according to renal stone status, using an analysis of variance (ANOVA) for continuous variables or a *χ*^2^ test for categorical variables.

^†^ Mean (SD).

^‡^ Median (interquartile range).

^§^ ≥ 20 g of ethanol per day.

### Comparison of microbial diversity among the renal stone groups

**Figure 2:**
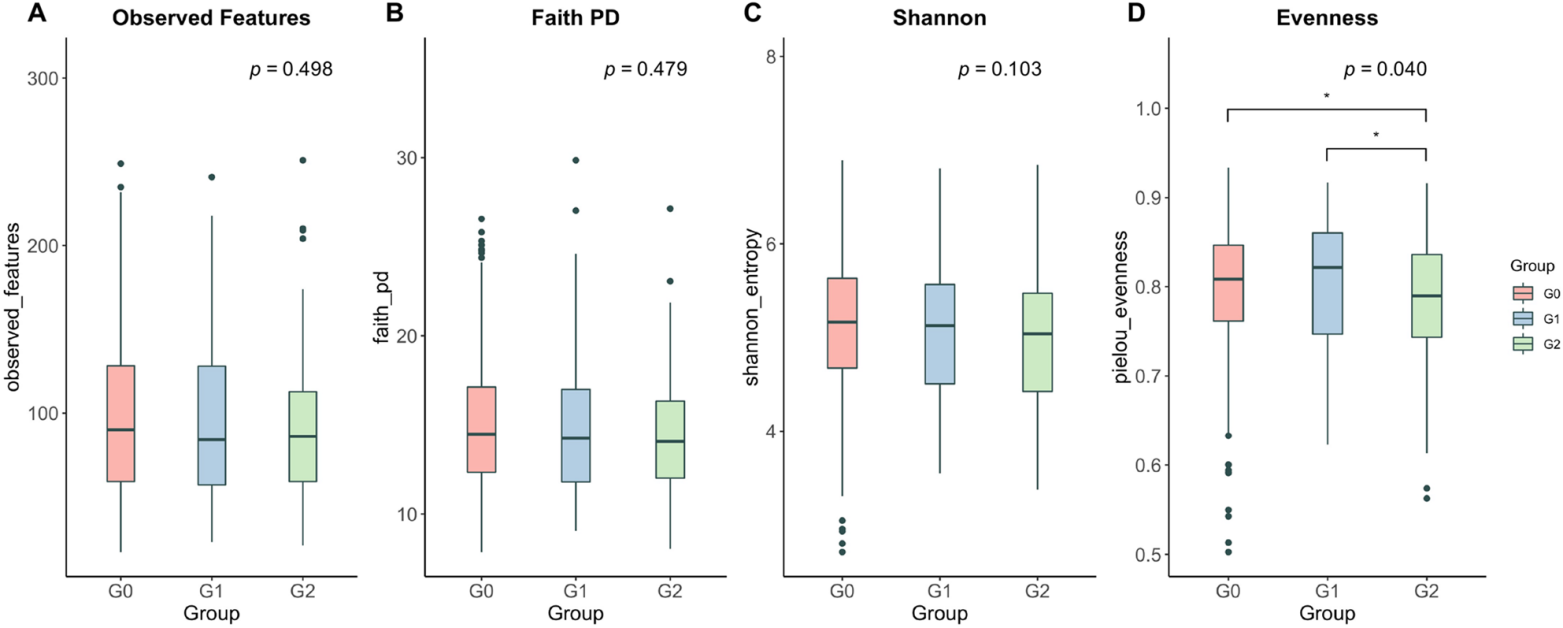
Alpha diversity of the microbiota among the renal stone groups. Boxplots representing alpha diversity include the observed features (**A**), Faith's phylogenetic diversity (**B**), Shannon's index (**C**), and Pielou's evenness (**D**) of the three groups. Statistics were calculated using a pairwise Kruskal–Wallis test among the three groups. The *p*-values were corrected using the Benjamini–Hochberg false discovery rate (FDR) (*p < 0.05). Boxes indicate the interquartile range (IQR) of the 25th to 75th percentiles. The median value is shown as a line within the box, and the whiskers extend to the most extreme value within 1.5 × IQR. Possible outliers are shown as dots. G0: no renal stones (control); G1: incidental renal stones; and G2: prevalent renal stones.

After rarefying the feature tables to 5011 reads per sample, the rarefaction curves showed that all groups tended to plateau, indicating that the biodiversity was adequately covered by the applied sequencing depth (Supplementary Fig. S1). We found a significant decrease in the microbial evenness in G2 compared with that in G0 (pairwise Kruskal–Wallis, *q* = 0.041) and G1 (*q* = 0.041) (Kruskal–Wallis in all three groups, *p* = 0.040, Fig. 2D). However, considering the richness, the number of observed features (*p* = 0.498, Fig. 2A), phylogenetic diversity (*p* = 0.479, Fig. 2B), and Shannon’s index (*p* = 0.103, Fig. 2C) were not significantly different among the three groups, although all indices showed the lowest median in G2. For the β-diversity analysis, there were no significant differences in the fecal bacterial communities among the three groups (Supplementary Fig. S2).

### Abundance of fecal bacterial communities according to the renal stone groups

To better understand how the microbial community composition fluctuated with renal stone incidence, we examined which organisms were present at different taxonomic levels and their relative abundances.

**Figure 3:**
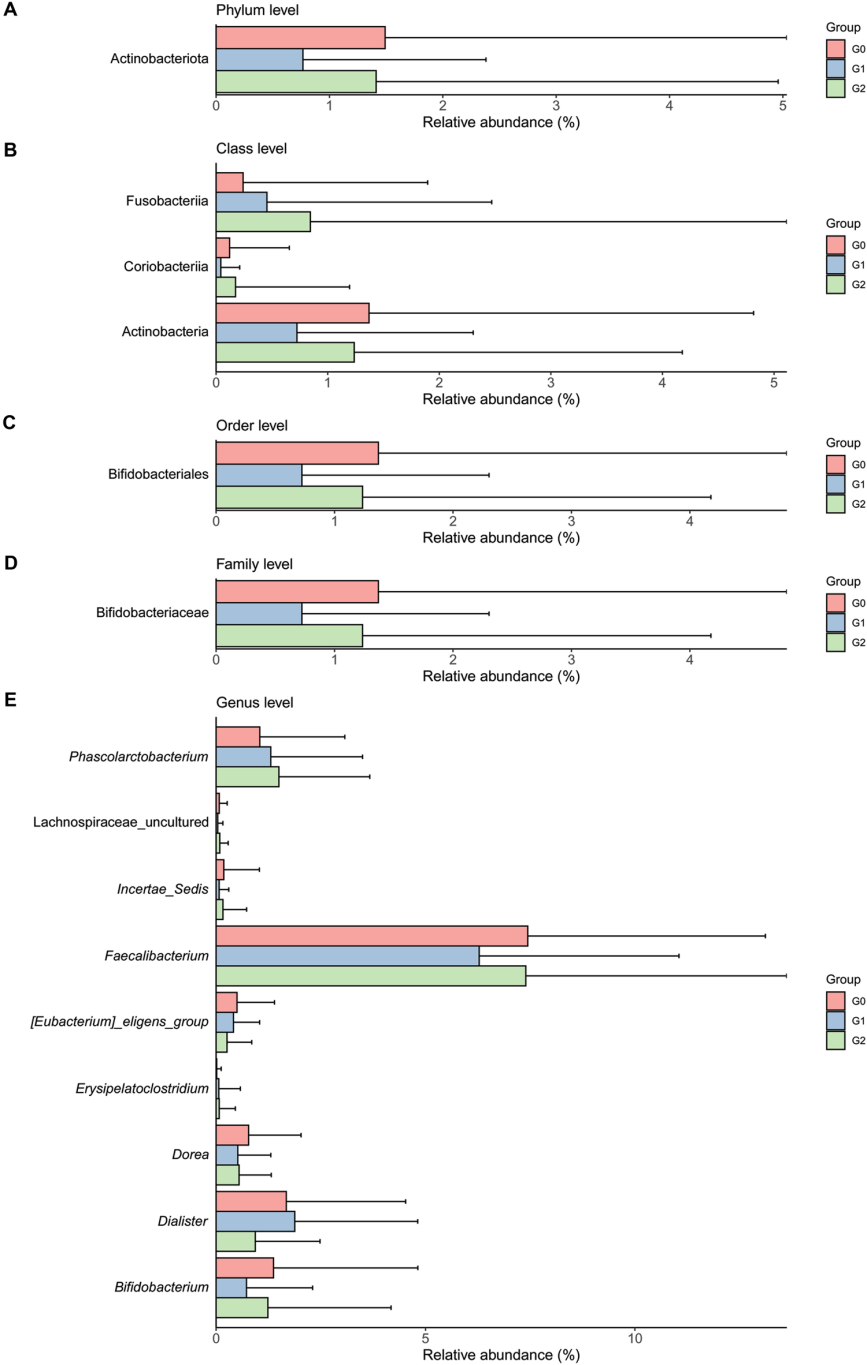
Bar plot showing the relative abundance of the taxa that differed significantly among the renal stone groups at the (**A**) phylum, (**B**) class, (**C**) order, (**D**) family, and (**E**) genus levels. Upper error bars represent the standard deviation. G0: no renal stones (control); G1: incidental renal stones; and G2: prevalent renal stones.

We first used ANCOM, which has a low false-positive rate and allows covariate adjustment^24,31^, and then used MaAsLin to ensure reproducibility of the results (Table 2). After adjusting some of the covariates, including sex, age, and BMI, we found that G1 had a significantly reduced abundance of the phylum Actinobacteria [ANCOM, *W* = 14; MaAsLin, exponentiated (exp) coefficient = 0.74; *p* = 0.01], the classes Actinobacteria [*W* = 15; exp(coefficient) = 0.80; *p* = 0.03] and Coriobacteriia [*W* = 14; exp(coefficient) = 0.81; *p* = 0.01], the order Bifidobacteriales [*W* = 32; exp(coefficient) = 0.80; *p* = 0.03], and the family Bifidobacteriaceae [*W* = 54; exp(coefficient) = 0.80; *p* = 0.03] compared to G0. According to the ANCOM results (*W* = 14), the class Fusobacteriia was more abundant in G2 than in G0. In addition, the MaAsLin results showed that Fusobacteriia was more abundant in G1 [exp(coefficient) = 1.16; *p* = 0.04] and G2 [exp(coefficient) = 1.25; *p* = 4.6 × 10^–3^] than in G0. At the genus level, the relative abundances of *Dorea*, *Incertae sedis*, *Bifidobacterium*, uncultured genus belonging to Lachnospiraceae, and *Faecalibacterium* were reduced by 10% (coefficient = –0.11) to 22% (coefficient = –0.25) in G1 compared to those in G0. We also found that the abundances of the genera *Eubacterium eligens group*, *Dialister*, *Phascolarctobacterium*, and *Erysipelatoclostridium* differed significantly between G0 and G2 (*W* > 170). The *Eubacterium eligens group* and *Dialister* were less abundant, while *Phascolarctobacterium* was more abundant in G2 than in G0. We observed that there were no significant differences in the composition of gut microbiota between G1 and G2. Notably, compared to G0, there were no shared significant taxa between G1 and G2, except for the class Fusobacteriia, in the MaAslin results. Figure 3 shows the relative abundance of the taxa represented in Table 2.

**Table 2:** Comparison of the microbial relative abundances between pairwise groups according to renal stone status at the phylum, class, order, family, and genus levels.

Taxonomic assignment	*W*^b^ (coefficients^c^)		
	G0^†^ vs. G1	G0^†^ vs. G2	G1^†^ vs. G2
p__Actinobacteriota	14 (–0.30*)	1 (**–**0.22)	0
p__Actinobacteriota; c__Actinobacteria	15 (–0.22*)	0 (**–**0.16)	0
p__Actinobacteriota; c__Coriobacteriia	14 (–0.21*)	0 (**–**0.15)	0
p__Fusobacteriota; c__Fusobacteria	7 (0.15*)	14 (0.22**)	0
p__Actinobacteriota; c__Actinobacteria; o__Bifidobacteriales	32 (–0.23*)	0 (**–**0.17)	0
p__Actinobacteriota; c__Actinobacteria; o__Bifidobacteriales; f__Bifidobacteriaceae	54 (–0.23*)	0 (**–**0.17)	0
p__Firmicutes; c__Clostridia; o__Lachnospirales; f__Lachnospiraceae; g__*[Eubacterium]_eligens_group*	0 (**–**0.05)	230 (–0.33**)	0
p__Firmicutes; c__Clostridia; o__Lachnospirales; f__Lachnospiraceae; g__*Dorea*	210 (–0.25**)	0 (**–**0.14)	0
p__Firmicutes; c__Clostridia; o__Oscillospirales; f__Ruminococcaceae; g__*Incertae_Sedis*	206 (–0.25**)	0 (0.03)	0
p__Firmicutes; c__Negativicutes; o__Veillonellales-Selenomonadales; f__Veillonellaceae; g__*Dialister*	0 (0.05)	201 (–0.28*)	0
p__Actinobacteriota; c__Actinobacteria; o__Bifidobacteriales; f__Bifidobacteriaceae; g__*Bifidobacterium*	190 (–0.23*)	0 (**–**0.17)	0
p__Firmicutes; c__Negativicutes; o__Acidaminococcales; f__Acidaminococcaceae; g__*Phascolarctobacterium*	0 (0.13)	176 (0.31*)	0
p__Firmicutes; c__Bacilli; o__Erysipelotrichales; f__Erysipelatoclostridiaceae; g__*Erysipelatoclostridium*	0 (0.02)	171 (0.20*)	0
p__Firmicutes; c__Clostridia; o__Lachnospirales; f__Lachnospiraceae; g__uncultured	170 (–0.22**)	0 (0.00)	0
p__Firmicutes; c__Clostridia; o__Oscillospirales; f__Ruminococcaceae; g__*Faecalibacterium*	159 (–0.11*)	0 (**–**0.07)	0

p__: phylum, c__: class, o__: order, f__: family, g__: genus, s__: species, G0: no renal stone group, G1: incidental renal stone group, G2: prevalent renal stone group.

^a^Number of phyla, 15; classes, 23; orders, 50; families, 89; genera, 260.

^b^*W* = X for taxon k, then *H*_*0k*_ is rejected X times. The W statistics for the significantly different taxa relative to over 60% of the other taxa at each taxon level are represented in bold.

^c^Coefficients for the log-transformed relative abundance of each taxon in the linear model adjusted for age, sex, and BMI using MaAsLin2 on pairwise comparisons between groups. * *p* < 0.05, ** *p* < 0.01.

^†^Group used as the reference group in the regression model.

**Figure 4:**
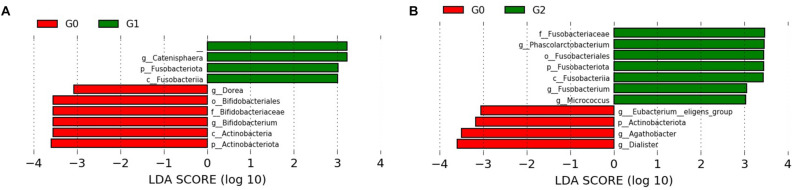
Linear discriminant analysis effect size (LEfSe) analysis identified the most differential microbiota between G0 and G1 (**A**), and G0 and G2 (**B**). The LDA score also indicates the effective size and ranking of each differentially abundant taxon (LDA score > 3.0; alpha value *p* < 0.05). Plots were depicted using LEfSe from the Galaxy platform of the Huttenhower lab. G0: no renal stones (control); G1: incidental renal stones; and G2: prevalent renal stones.

Based on the results of diversity and taxonomic analysis, we performed LEfSe analysis to detect potential bacterial markers that most likely explain the differences among the renal stones groups by coupling standard tests for statistical significance with additional tests encoding biological consistency and effect relevance^25^. Comparisons of G0 vs. G1 and G0 vs. G2 identified 10 and 11 taxa with linear discriminant analysis scores of > 3, respectively (Fig. 4). The LEfSe results confirmed the significant enrichment of Actinobacteria, Bifidobacteriales, *Dorea* spp., and *Bifidobacterium* spp*.* in G0 compared to that in G1 (Fig. 4A). We also found that Fusobacteria and *Catenisphaera* spp. were more abundant in G1 than in G0. A significantly higher abundance of the genera *Phascolarctobacterium*, *Fusobacterium*, and *Micrococcus* was noted in G2 than in G0 (Fig. 4B). The significantly higher abundance of the genera *Eubacterium eligens group* and *Dialister* in G0 than in G2 was also confirmed in the LEfSe results.

### Predicted metabolic pathways

**Figure 5:**
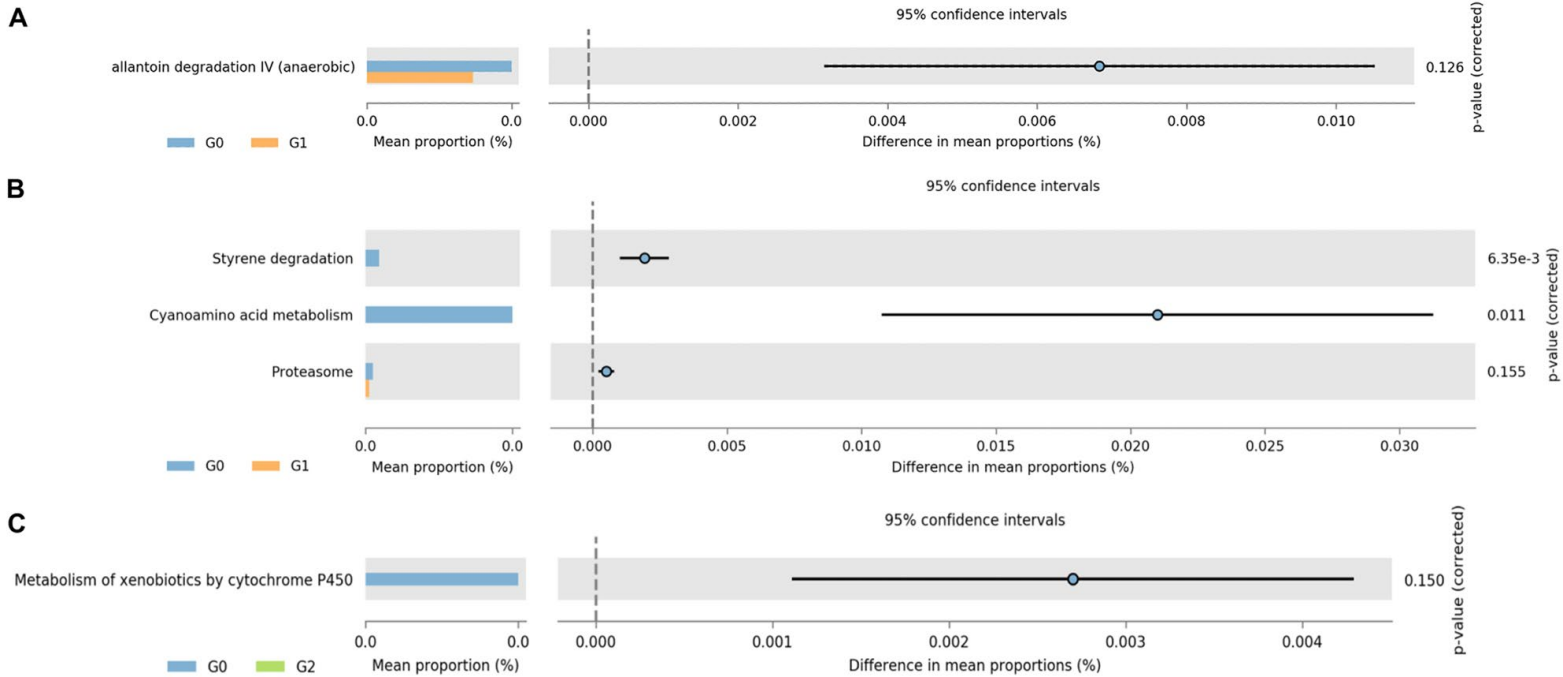
Abundances of the predicted functional pathways were different among the renal stone groups. Extended error bar plots show the significantly different MetaCyc (**A**) and KEGG (**B**) pathways between G0 and G1. *Metabolism of xenobiotics by cytochrome P450* of the KEGG pathway was less abundant in G2 compared to that in G0 (**C**). Bar plots on the left display the mean proportion of each pathway. Dot plots on the right show differences in the mean proportions between the two indicated groups using the *p*-value. In STAMP, differences in the abundances between the two groups were compared using White's non-parametric t-test. The *p*-values corrected using the Benjamini–Hochberg FDR are shown on the right. G0: no renal stones (control); G1: incidental renal stones; and G2: prevalent renal stones.

For a better understanding of the function of renal stone-associated bacteria, we inferred the predictive pathways using the MetaCyc and KEGG databases of PICRUSt2. Figure 5 shows the statistically significant (FDR *q* < 0.05) and suggestive (FDR *q* < 0.25) metabolic pathways and how they contributed differently to each of the three groups according to renal stone status. Among the pathways, a MetaCyc pathway related to *allantoin degradation IV (anaerobic)* and three KEGG pathways related to *styrene degradation, cyanoamino acid metabolism, and proteasome* exhibited significant differences between G0 and G1, with decreased pathways in the G1 compared to those in G0 (Fig. 5A, B). A KEGG pathway related to *the metabolism of xenobiotics by cytochrome P450* was significantly less abundant in G2 than in G0 (Fig. 5C). There was no statistically significant difference in the pathways between the G1 and G2 groups.

## Discussion

In this study of 915 participants with repeated ultrasonography examinations, the incidental and prevalent stone groups showed an altered gut microbiota composition and functionality, compared with no stone group at both the initial and follow-up visits. While most previous studies comparing groups with and without nephrolithiasis have predominantly focused on *O. formigenes*, our findings indicate that other gut microbiota might be involved in nephrolithiasis risk. Moreover, our study differentiated incident from prevalent stones, with relatively large sample sizes in each group, and explored the differences in the gut microbiome and metabolic pathways.

Gut microbiome alpha-diversity has been linked to human health, with lower levels of diversity associated with several chronic diseases^32^. A reduction in diversity is called microbial imbalance or dysbiosis. We found that the gut microbiome of the prevalent stone groups showed lower evenness than those of the other groups, reflecting changes in the abundance of some specific taxa.

Despite the essential role of *O. formigenes* in oxalate formation, recent human studies using 16S or metagenomic techniques have reported no differences in the gut species between healthy control groups and kidney stone groups^10–14^. Our results were consistent with those of the previous reports that found significant differences in the abundances of other taxa as opposed to *Oxalobacter* between the control group and the renal stone group. We found that *Bifidobacterium* was more abundant in no stone group than in incidental stone group. *Bifidobacterium* is also known to effectively degrade oxalate, albeit somewhat less effectively (11–68%) than *O. formigenes* (98%)^33^. Tavasoli et al. suggested that although some *Lactobacillus* and *Bifidobacterium* strains have oxalate-degrading potential, they may not be among the major oxalate-degrading bacteria of the gut microbiome. Recently, Denburg et al. reported that oxalate-degrading bacterial taxa, including *Eggerthella lenta* and several *Lactobacillus* species, were decreased in kidney stone formers^34^.

We also found that the abundances of *Dorea*, *Incertae sedis*, and *Faecalibacterium* were lower in incidental stone group than in no stone group. Additionally, compared with no stone group, the incidental stone group showed high abundances of Fusobacteria, *Phascolarctobacterium,* and *Erysipelatoclostridium*, and lower abundances of *Eubacterium eligens group* and *Dialister*. The association between these taxa and kidney stones has been reported in previous studies^10–12,14^. These observations imply that the gastrointestinal-renal axis may not solely rely on a single player or a limited number of species^11,14^. Ticinesi et al.^14^ suggested that oxalate-degrading activity may be shared by various taxa, influencing each other in a complex metabolic network.

Interestingly, we observed a lower abundance of *Faecalibacterium* in incidental stone group than in no-stone group, while there was no difference in abundances between prevalent stone and no-stone groups. A notable reduction in *Faecalibacterium prausnitzii* abundance has been reported in kidney stone groups in previous studies^11,14,35^. This bacterium is a principal manufacturer of short-chain fatty acids (SCFAs) within the human intestinal microbiome^11,14^. Recently, Liu et al.^36^ reported on the relationship between the gut microbiota and SCFAs regarding nephrolithiasis. They found that the abundance of pathways involved in SCFA production was positively correlated with the level of bacteria in the gut microbiota in the no-stone control group, but not in groups with kidney stones, which indicated that the gut microbiota of no-stone controls may tend to produce more SCFAs than that of nephrolithiasis patients. Moreover, the proportion of renal crystals was reduced after the administration of acetate, propionate, or butyrate in a rat model, which indicated that SCFAs could effectively prevent the formation of renal calcium oxalate crystals^36^. On the other hand, *Dialister,* which is also an SCFA-producing bacterium, was only depleted in prevalent stone group. We observed that there were no bacteria that showed significant differences in both the incidental stone and prevalent stone groups, when compared to no-stone group, and were associated with either incidental stone or prevalent stone group. This may be due to differences in the lifestyles of the incidental stone and prevalent stone groups. It is possible that patients with prevalent renal stones can alter their lifestyle behaviors, which may affect the gut microbiota.

The potential gastrointestinal-renal axis in patients with nephrolithiasis was recently proposed by Ticinesi et al.^37^. These authors placed *O. formigenes* at the heart of a bacterial complex that was engaged in the breakdown of oxalate and avoidance of hyperoxaluria. In contrast to the more straightforward axial theory, which emphasizes the role of *O. formigenes* alone, these authors postulated the involvement of additional taxa that had oxalate-metabolizing properties, together with taxa that were triggered by the presence of oxalate and worked synergistically to break down salt, thus diminishing oxalate absorption.

Interestingly, the 16S data suggested that several metabolic pathways were related to renal stone formation. As allantoin is the final covert material created by uricase, allantoin degradation could be related to solubility. Therefore, allantoin degradation could increase uric acid contents^38^. Regarding styrene degradation based on microbiological activity, the citrate cycle can be affected^39^. Indeed, hypocitraturia or low urinary citrate excretion is a common feature in patients with nephrolithiasis. The pathway involved in the proteasome includes an interaction between calcium oxalate crystals and renal tubular epithelial cells^40^. Furthermore, the metabolism of xenobiotics by cytochrome P450 can be affected by the human gut microbiome, contributing to various diseases, including kidney stones^41^.

This study incorporated a large population and investigated the association between the gastrointestinal microbiome and nephrolithiasis. However, the relevance of these results to current or potential clinical practices is not immediately apparent. In view of the multifaceted nature of the gut microbiome, further research is necessary to demonstrate the effectiveness of interventions aimed at restricting the absorption of oxalate across the intestinal barrier. Our study did not incorporate 24 h urine analysis or stone chemical analysis. Information on stone size and specific stone type, determined by detailed image or composition analyses, was also unavailable. We used abdominal ultrasonography to diagnose nephrolithiasis, although unenhanced computed tomography (CT) is considered a more accurate reference method. A recent study comparing ultrasonography and CT reported a fair sensitivity of 70.0% and specificity of 94.4% for detecting nephrolithiasis^42^. Even though ultrasonography can be both appropriate and feasible in a large population study without radiation hazard, we cannot exclude misclassification of nephrolithiasis determined using ultrasonography. Furthermore, seasonal variations were not considered in this study. It is possible that participants in the incidental stone group had prior stone episodes but no stones on the baseline ultrasonography during the same season. Moreover, among the prevalent stone patients (*n* = 86), 76 (88.4%) had at least one follow-up visit, 50 (65.8%) were diagnosed with nephrolithiasis, and 26 (34.2%) had no stones detected at follow-up. Those with prevalent nephrolithiasis without stones at follow-up may represent a false positive result for nephrolithiasis or stone passage during follow-up. Unfortunately, detailed information on stone treatment or stone passage was not available, limiting our ability to differentiate between the two conditions. Additionally, subjects who had passed stones spontaneously and had no stones visible on ultrasound might have been misclassified as no stone group. Thus, no differentiation between symptomatic and asymptomatic stones and misclassification of stone formers might limit the implication of our findings. Another limitation of our study is that we only obtained the baseline fecal samples of participants without microbiome data at follow-up, which restricted our ability to examine the relationship between changes in the fecal microbiome and renal stone status over time. Further analysis including serial data of gut microbiota during follow-up periods is required to elucidate the longitudinal association between microbiome and renal stone changes over time. Although diet is a key factor in shaping the gut microbiota, the gut microbiome profile in individuals susceptible to renal calculi does not appear to be affected by diet in our study. Ticinesi et al.^14^ reported that dietary habits are apparently not involved in the nephrolithiasis-associated abnormalities of the gut microbiota composition, with the only possible exception being calcium intake. Moreover, additional elements may alter the constitution of the bacteria, which in turn may have a bearing on the pathophysiology of renal stone disease. Finally, our study was based on 16S rRNA gene sequencing, which provides limited information about bacterial genes and their functions. Whole metagenomic sequencing could expand our understanding of the strains associated with renal stones, their genes and functions, and metabolic pathways. Additionally, concordant analysis including the urinary and fecal microbiome could explain the relationship between the gut microbiome and nephrolithiasis more clearly^43,44^. Additional studies are required to discern the way in which the composition and purpose of the microbiome may impact therapy outcomes and influence renal stone disorders.

Compared with healthy controls, the incidental and prevalent renal stone groups exhibited an altered gut microbiota composition that could contribute towards nephrolithiasis physiopathology. Besides the known oxalate degradation pathway, other functional pathways inferred from taxonomic profiles have been suggested, which require further investigation in the future.

## Supplementary Information

Supplementary Information.

## Data Availability

The datasets in the current study are available from the corresponding author upon reasonable request, and the raw 16S rRNA gene sequence data can be found in a public repository: the Clinical & Omics Data Archive (CODA) at the Korea National Institute of Health (accession number R000635; http://coda.nih.go.kr/coda/coda/search/omics/genome/selectSearchOmicsGenomePop/R000635.do).
