# Supplementary material for: Gut microbiota and the prevalence and incidence of renal stones
Source: Sci Rep. 2022 Mar 8;12:3732. doi: 10.1038/s41598-022-07796-y (PMC8904816; doi:10.1038/s41598-022-07796-y)
Supplement: Supplementary file 1 — Supplementary Information. [file 41598_2022_7796_MOESM1_ESM.docx]

**Supplementary data**

**Gut microbiota and the prevalence and incidence of renal stones**

Han-Na Kim, Jae Heon Kim, Yoosoo Chang, Dongmin Yang, Kwan Joong Joo, Young-Sam Cho, Heung Jae Park, Hyung-Lae Kim, and Seungho Ryu

**Supplementary Methods**

*Characteristics of study subjects and the selection process*

This study was performed as a part of the Kangbuk Samsung Health Study, a cohort study of Koreans who underwent comprehensive annual or biennial examinations at the Kangbuk Samsung Hospital Healthcare Screening Center in South Korea ^1^. In Korea, the Industrial Safety and Health Law requires employees to participate in annual or biennial health examinations, which are offered free of charge. More than 80% of the study participants or their spouses were employees of various companies and local government organizations. The remaining participants were individuals who voluntarily underwent screening examinations. The present study included 1463 Korean adults aged 23 to 78 years who agreed to participate in this study and provided stool samples at Kangbuk Samsung Hospital Healthcare Screening Center ^2^. These participants were recruited from among those who underwent annual or biennial examinations between June 2014 and September 2014. Written informed consent was obtained from all participants included in this study. Fecal samples were obtained only at baseline. In a health checkup program, abdominal ultrasound and an annual or biennial follow up are routinely performed. Each participant was followed up from the baseline examination until the last examination regardless of stone status, and the median follow-up duration (interquartile range) was 4.6 (3.6-5) years in G0 group; 4.7 (1.9-5.0) years in G1 group; and 4.8 (3.9-5.1) years in G2 group. The follow-up duration was similar across all groups. Among the 1456 participants with available information on ultrasound, 121 (8.1%) had nephrolithiasis based on ultrasound at baseline, before applying the exclusion criteria (Figure 1). After excluding participants based on the following criteria: use of antibiotics or probiotics within six weeks prior to enrollment (n = 72), use of antacids (n = 32), use of anti-diabetic medication (n = 53), history of malignancy (n = 52), history of kidney disease (n = 9), history of gout (n = 24), history of liver cirrhosis or findings of liver cirrhosis on ultrasound (n = 3), missing information on ultrasound (n = 7), and samples with fewer than 5000 sequences per sample (n = 90), 86 participants (7.5%) out of 1148 participants had prevalent nephrolithiasis. Out of 1062 participants free of nephrolithiasis at baseline, 233 participants with no follow-up by December 31, 2019 were further excluded for examining the prospective association between gut microbiome and incident nephrolithiasis. Ultimately, 97 incident nephrolithiasis cases were identified during follow-up.

**Supplementary Table S1.** Nutrient characteristics of study participants according to renal stones.

| Nutrients | G0 (n = 534) | G1 (n = 68) | G2 (n= 62) | *p* |
| --- | --- | --- | --- | --- |
| Total energy, kcal/day | 1425.6 ± 634.7 | 1365.5 ± 663.1 | 1500.4 ± 665.1 | 0.487 |
| Total carbohydrate, g/day | 237.5 ± 107.1 | 234.9 ± 119.8 | 254.5 ± 111.3 | 0.487 |
| Total fat, g/day | 29.2 ± 19.8 | 25.0 ± 16.9 | 29.4 ± 18.9 | 0.230 |
| Total protein, g/day | 49.6 ± 25.9 | 45.8 ± 23.3 | 50.59 ± 24.3 | 0.464 |
| Cholesterol, mg/day | 178.4 ± 152.3 | 154.8 ± 115.2 | 181.1 ± 127.1 | 0.444 |
| Fiber, g/day | 3.7 ± 2.3 | 3.5 ± 2.3 | 3.8 ± 1.8 | 0.702 |
| Folate, mg/day | 151.8 ± 102.2 | 137.5 ± 94.4 | 153.2 ± 81.2 | 0.524 |
| Total phosphorus, mg/day | 720.4 ± 350.9 | 666.3 ± 318.0 | 754.9 ± 371.6 | 0.333 |
| Retinol, ug/day | 73.6 ± 61.7 | 62.4 ± 47.2 | 81.3 ± 64.3 | 0.194 |
| Total sodium, mg/day | 1650.5 ± 1082.3 | 1505.4 ± 1072.6 | 1583.6 ± 878.0 | 0.536 |
| Total Vitamin A, ug/day | 336.3 ± 259.0 | 318.2 ± 232.9 | 336.0 ± 197.8 | 0.854 |
| Vitamin B1, mg/day | 0.850 ± 0.468 | 0.779 ± 0.422 | 0.865 ± 0.425 | 0.462 |
| Vitamin C, mg/day | 70.7 ± 58.9 | 61.4 ± 60.6 | 74.8 ± 44.8 | 0.370 |
| Total calcium, mg/day | 314.1 ± 205.8 | 257.5 ± 167.1 | 334.25 ± 260.2 | 0.069 |

Data are presented as mean (± SD).

*P*-values were calculated using the analysis of variance (ANOVA) among groups.

G0: no renal stone group, G1: incidental renal stone group, G2: prevalent renal stone group

**Supplementary Figure S1.** **Rarefaction plots based on alpha diversity metrics.** The number of observed features indicated that 5,011 sequences per sample are sufficient for capturing the alpha diversity of microbial communities in the three groups by renal stones. The x-axis shows the number of sequences per sample. Rarefaction curves construction (10 replicates/depth) were performed using the “diversity alpha-rarefaction" plugin QIIME2. G0: no renal stone group, G1: incidental renal stone group, G2: prevalent renal stone group


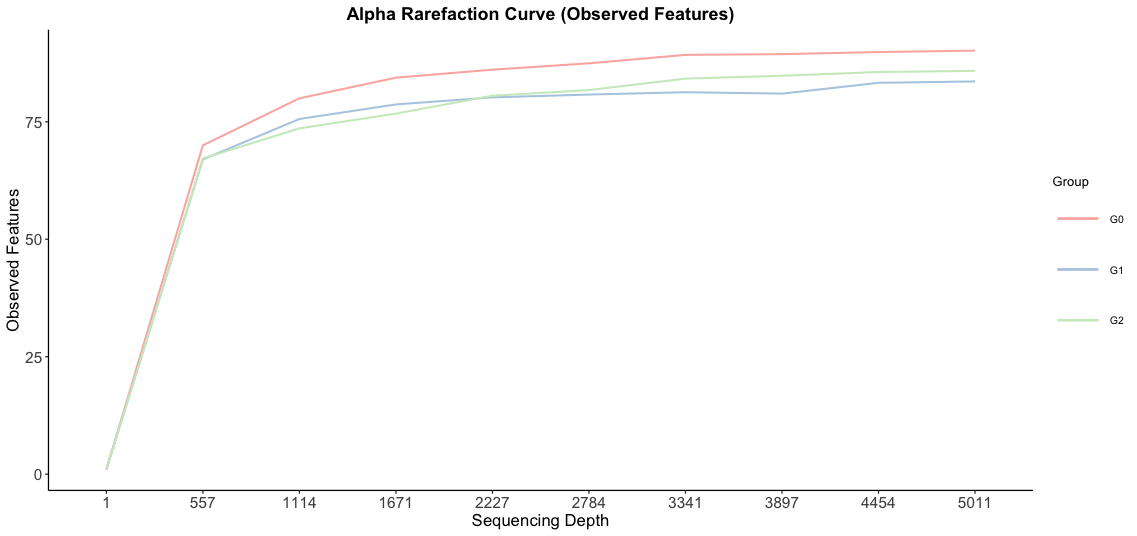


**Supplementary Figure S2.** **Beta-diversity of microbial taxa.** Principal Coordinate Analysis (PCoA) plots representing the β-diversity for (A) Unweighted UniFrac distance, (B) Weighted UniFrac distance, (C) Jaccard distance, and (D) Bray-Curtis dissimilarity comparing subjects by renal stones. Each point represents an individual subject. Statistics were calculated using pairwise PERMANOVA with 999 permutations. Ellipses represent the 95% confidence interval for each group. G0: no renal stone group, G1: incidental renal stone group, G2: prevalent renal stone group

**Supplementary References**

1 Chang, Y. *et al.* Metabolically Healthy Obesity and Development of Chronic Kidney Disease: A Cohort Study. *Ann Intern Med* **164**, 305-312, doi:10.7326/m15-1323 (2016).

2 Kim, H. N. *et al.* Correlation between gut microbiota and personality in adults: A cross-sectional study. *Brain Behav Immun* **69**, 374-385, doi:10.1016/j.bbi.2017.12.012 (2018).
